# What's on your plate? Collecting multimodal data to understand commensal behavior

**DOI:** 10.3389/fpsyg.2022.911000

**Published:** 2022-09-30

**Authors:** Eleonora Ceccaldi, Radoslaw Niewiadomski, Maurizio Mancini, Gualtiero Volpe

**Affiliations:** ^1^Casa Paganini-InfoMus, Dipartimento di Ingegneria, Bioingegneria, Robotica ed Ingegneria dei Sistemi (DIBRIS), University of Genoa, Genoa, Italy; ^2^Dipartimento di Psicologia e Scienze Cognitive, University of Trento, Rovereto, Italy; ^3^Department of Computer Science, Sapienza University of Rome, Rome, Italy

**Keywords:** datasets, commensality, Multimodal Human-Food Interaction, social signal processing, activity recognition

## Abstract

Eating is a fundamental part of human life and is, more than anything, a social activity. A new field, known as *Computational Commensality* has been created to computationally address various social aspects of food and eating. This paper illustrates a study on remote dining we conducted online in May 2021. To better understand this phenomenon, known as *Digital Commensality*, we recorded 11 pairs of friends sharing a meal online through a videoconferencing app. In the videos, participants consume a plate of pasta while chatting with a friend or a family member. After the remote dinner, participants were asked to fill in the Digital Commensality questionnaire, a validated questionnaire assessing the effects of remote commensal experiences, and provide their opinions on the shortcomings of currently available technologies. Besides presenting the study, the paper introduces the first Digital Commensality Data-set, containing videos, facial landmarks, quantitative and qualitative responses. After surveying multimodal data-sets and corpora that we could exploit to understand commensal behavior, we comment on the feasibility of using remote meals as a source to build data-sets to investigate commensal behavior. Finally, we explore possible future research directions emerging from our results.

## 1. Introduction

This work aims at exploring remote commensal experiences. Here, we present a study on users' experience of online meals, measured through facial expressions and questionnaires, and a data-set of online meals that we collected for this purpose, the Digital Commensality Data-set. The goal of the work is two-fold: to investigate users' experience of Digital Commensality through quantitative and qualitative measures and to collect multimodal data, thus exploring the feasibility of a remote data collection.

Food and eating-related activities, such as cooking, drinking, and sharing food, are, in fact, extremely interesting, rich in affective and social cues, and eating, research says, is inherently social (Simmel, 1997). Nowadays, eating is tightly linked with technology, which affords the augmentation of meals, allows the creation of multisensory food experiences, and can foster commensality even in loneliness by providing artificial dining companions or the possibility to share meals remotely (Mancini et al., 2020). According to research (Ceccaldi et al., 2020), people tend to eat with others online for the same reasons they eat with others offline: to gain a sense of togetherness and belonging, to feel less lonely, and because sharing a meal with someone makes them appreciate their food more. Nonetheless, current technologies do not seem to provide entirely satisfactory commensal experiences. The role of technology in eating activities has recently gained more interest in research. In the field of Human-Computer Interaction, the emerging research area of Multimodal Human-Food Interaction (see Altarriba et al., 2019; Deng et al., 2021) aims to investigate the relationship between humans, food, and technology. This relationship can take different forms, and several various topics have been identified by Velasco et al. (2022), namely: data collection, psychological mechanisms underlying human-food interaction such as crossmodal effects (Mathiesen et al., 2022), design studies and frameworks, augmentation and application, such as health, entertainment, and commensality. Regarding the latter, we contributed to creating the research area known as Computational Commensality to target this specific form of human-food interaction.

Niewiadomski et al. (2019) coined the term *Computational Commensality* to describe a new area at the intersection between Human-Computer Interaction, Computer Science, and Psychology. At the core of Computational Commensality lies the idea that food is a social phenomenon and that computational models aiming to address, augment, analyze or recognize food and eating-related behavior should consider this social dimension. Commensality, in a sense, is seen as a non-verbal social signal, with food and food-related activities being treated as sources of information to understand social interaction. Being at the intersection of psychology and technology, one of the fundamental interests in Computational Commensality is using technology to augment, assist, and improve mealtimes. Therefore, researchers have proposed technologies to, e.g., augment taste and flavor (Velasco et al., 2018), create robots acting as meal companions (Fujii et al., 2021), and foster playful food-related interactions. What is more, Computational Commensality has investigated ways in which technology can afford remote commensality, allowing commensals to share meals while physically apart. The term *Digital Commensality* indicates different scenarios, from sharing a meal through Skype (or similar technologies)—also referred to as Skeating (Spence, 2017), to Mukbang. In this recent trend, people watch somebody else video-streaming their meal without actually interacting (Kircaburun et al., 2021). Digital Commensality is, in fact, a sub-topic in Computational Commensality focusing on meals shared with or through digital technologies (Mancini et al., 2020).

In this paper, we investigate remote meals through the lenses of Computational Commensality, exploring users' experience of Digital Commensality through a computational approach. Specifically, we address three research questions. First, we analyze participants' responses to the Digital Commensality questionnaire, investigating whether, after sharing a meal online, participants perceive a sense of togetherness and belonging that, according to previous studies (Ceccaldi et al., 2020), are among the reasons to share a meal online (Research Question 1). Also, we observe participants' responses to open-ended questions to check whether they identify major shortcomings of currently available Digital Commensality experiences and future directions for Digital Commensality technologies (Research Question 2). In addition, we explore whether we can detect participants' experiences of commensality through automatic analysis of facial features (Research Question 3). What is more, as a result, we present the first Digital Commensality Data-set: a data collection on pairs sharing a meal online. Despite the creation of social interaction data-sets being a common approach in research, collecting data on (digital) commensal behavior poses, we argue, specific challenges. First and foremost, to ensure ecological validity, such interactions need to be observed where they would naturally occur, i.e., at home instead of a laboratory setting, resulting in a lack of control. Second, this approach might lead to privacy issues, with the need to record participants through their personal computers. In this paper, we illustrate the methodology we adopted to create the data-set and comment on the feasibility of collecting data-sets remotely.

The paper is organized as follows: in Section 2, we present the idea of addressing Commensality through multimodal data-sets and provide examples of data-sets and corpora that we could leverage for commensality research; Section 3 introduces the Digital Commensality Data-set and the data-collection methodology; Section 4 describes the analysis we carried out and provides insight on possible ways to exploit our data for social interaction research. Section 5 discusses our results while the Conclusion section ends the paper.

## 2. Related work

To better introduce our approach, this section illustrates other data-sets on food and eating-related activities. With this aim, we searched for papers in Multimodal Human-Food Interaction describing data-sets that were meant at or could be leveraged for Computational Commensality. Here, we mention papers meeting at least one of the following criteria: (a) containing food items or drinks, for instance, images of food items, (b) displaying food-related activities, such as cooking, chopping, cutting, etc., and (c) including eating-related behavior, for example chewing, sipping, drinking. Sources were identified starting from Velasco et al. (2022), where a list of relevant workshops and journals is presented, and by looking at IEEE and ACM archives, searching for the following keywords: *food data-set, eating activity data-set, commensality data-set*. Although data-sets can have many different forms, such as dietary studies that were pivotal in starting commensality research (De Castro, 1994), here we focus on video, motion capture, image, and audio data-sets, as these forms of data-collection are closer, in methods and goals, to our study.

Capturing movements through videos and motion capture technologies is a widespread approach in Computational Science, affording a thorough measurement and observation of verbal and non-verbal signals. Moreover, data-sets may also be accompanied by self-report measurements provided by the participants portrayed in the videos, thus enriching the content with measures of their opinions, affective state, and so on. When it comes to food and eating related activities, such methodologies can help understand, observe, and measure both coarse and fine-grained movements needed to consume, prepare or even share food. For instance, Stein and McKenna (2013) created a data-set of manipulative gestures containing RGB videos and accelerometer data of people preparing mixed salads, and Rohrbach et al. (2012) presented a data-set of 65 fine-grained cooking activities captured through video and high-resolution images. Moreover, the CMU Multi-Modal Activity Database (De la Torre et al., 2009), collected at Carnegie Mellon's Motion Capture Lab, features recordings of participants cooking different recipes. In addition to cameras with different resolutions, researchers recorded cooking activities through microphones, motion-capture, internal measurement units, and wearable devices. Even if not specifically intended to target commensality, such data-sets can be fruitful sources to investigate the social aspects of eating. Hossain et al. (2020) proposed a data-set of people consuming meals in a laboratory setting, containing videos of participants eating a meal of their choice in groups of three. Although their data-set was aimed at automatically detecting chews and bites, it could be exploited by Computational Commensality studies as it features recordings from commensal scenarios. Besides providing data on specific instances of food-related behavior, data-sets-based studies also act as sources of inspiration, suggesting new ways to analyze and understand data. In a video-based study on bread consumption patterns, Miele et al. (2021) propose an approach called Temporal Dominance of Behavior, based on analyzing videos in terms of frequency, duration, sequence, and simultaneity of a given set of actions. Although, as they mention, adopting this approach to the study of commensality would require considering a different set of actions, their study nonetheless suggests an interesting way to investigate the social side of meals.

Regarding images, many data-sets exist, having different applications ranging from personalized diet-supporting apps to food production monitoring. Mainly, such data-sets are used to train and test food recognition and classification algorithms. These data-sets are often quite large, as studies can benefit from the many pictures people share on social media daily. However, such pictures are mainly egocentric ones captured with mobile devices (i.e., smartphones), usually of low quality, poor framing, often out of focus, blurry, and taken in low illumination. While we can find food and drink images in large, general use image data-sets, such as ImageNet (Russakovsky et al., 2015), containing hundreds of different object categories, data-sets have been explicitly proposed providing food images: the publicly available data-set Food101 by Bossard et al. (2014) contains 101,000 images and 101 food categories, such as: escargots, paella, risotto, or bibimbap. Similarly, the freely available UECFood256 data-set by Kawano and Yanai (2014) contains 256 different categories, while the UNIMIB2016 data-set by Ciocca et al. (2017) consists of more than a thousand tray images with multiple foods belonging to 73 classes. Interestingly, the latter includes the leftover images acquired after the meals (Ciocca et al., 2015). Interestingly, food data-sets may be paired with information on recipes (Chen et al., 2017) or ingredients (Bolanos et al., 2017), or with annotations indicating macro nutrients (Horne et al., 2019) or calories (Fromm et al., 2021).

What is more, data-sets have been proposed that accompany images with affective ratings: the OLAF (Open Library of Affective Foods) (Miccoli et al., 2016) contains food images and ratings created to study emotions toward food. The FoodCast Research Image Database (FRIDa) (Foroni et al., 2013) also provides affective ratings for food images and familiarity ratings.

Audio features should not be neglected in investigating Computational Commensality, as eating is a highly multisensory experience (Spence, 2017). Fortunately, some data-sets are available in the literature focusing on audio features: the iHEARu-EAT database, for instance, features recordings from 30 subjects eating 6 different kinds of food (Hantke et al., 2016), whereas the Eating Sound data-set proposed by Ma et al. (2020) includes audio from 20 different food types. Besides giving information on the kind of food that is consumed or on possible conversation topics, one could also exploit this modality to have a better picture of the commensal scenario. For instance, the CORSMAL Container Manipulation Data-set, proposed by Donaher et al. (2021), contains audio from the manipulation of different containers and includes two different kinds of manipulation: shaking and pouring.

As shown by the variety of available data-sets, which we only mention a few of, different angles, approaches and methodologies have been adopted to create data-sets to investigate food-related behavior. Often, these data-sets contain data from solo meals or, when commensal experiences are recorded, the social side of eating is often neglected. In the following section, we illustrate a data-set created to study commensal behavior and, more specifically, Digital Commensality in the form of remote meals.

## 3. The Digital Commensality data-set

The data analyzed in our study is part of the *Digital Commensality Data-set*, which consists of: (1) face Landmarks and Action Units (Ekman and Friesen, 1978), (2) qualitative responses on the Digital Commensality experience and suggestions for future commensal technologies, and (3) quantitative responses to the Digital Commensality questionnaire (Ceccaldi et al., 2020). We carried out the data collection in May 2021.

To the best of our knowledge, the data-set is the first one on remote meals. We asked participants to fill in a consent form upon agreeing to participate in the study. All participants consented to be audio and video recorded and the resulting recordings to be used by the researchers, along with their anonymized or aggregated data. Therefore, anonymized collected data is publicly available.^1^ Table 1 summarizes the content of the data-set, along with the availability of the different data sources.

**Table 1:** The Digital Commensality Data-set content description and availability.

**Data**	**Description**	**Available**
Video recordings	Recordings from participants' web cameras	No
Audio recordings	Recordings from participants' microphones	No
Face landmarks and action units	Anonymous data extracted through the OpenFace software	Yes
Audio transcripts	Anonymous transcription of the participants conversations	Yes
DC scores	Likert-item ratings of DC questionnaire items	Yes

### 3.1. Participants

In total, 22 volunteers took part in the recordings, 12 were females, and 10 males. Out of the 22 participants, 16 were between 18 and 24 years old, 3 between 25 and 29, and 3 were over 55. All participants were from Italy. Out of the 11 pairs, 3 were made up of colleagues, while 8 were best friends. When asked about their videocalls usage, most participants reported using videocalls every day or multiple times a week for work, but rarely for meeting friends or family.

### 3.2. Procedure

Upon they agreed to take part in the study with a friend or family member, we sent participants an email containing their unique IDs, the link to the first questionnaire (see Section 3.4), the instructions for setting up their table with a computer, a consent form to be filled out before taking part in the data collection, and the link to an online video call with the experimenter. After logging in, the experimenter greeted them and informed them about the protocol and the option to drop out at any point if they desired to do so. After checking the table and computer setup, the experimenter invited the participants to start eating, telling them to talk freely and suggesting them a topic to start chatting, in case they did not have any topic they would prefer to speak about (e.g., what meal they would order in an ideal best restaurant, in which they could order whatever meal they could think of). Then, the experimenter muted his mic and turned off his webcam. We gave no constraints to the participants regarding meal duration: they were invited to eat for as long as needed and to signal to the experimenter when their meal was over. When that happened, the experimenter would join the call again to answer participants' questions and ask them to complete the final questionnaire, described in Section 3.4. Participants were all volunteers recruited among the experimenters' friends and relatives.

### 3.3. Set-up

**Figure 1:**
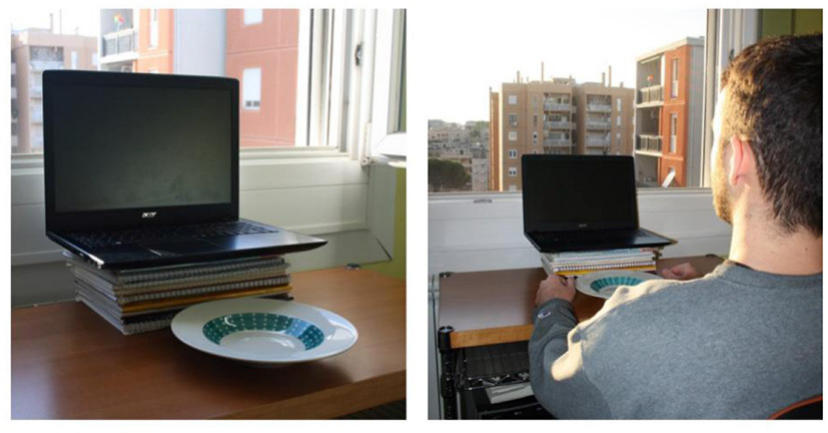
The data collection technical setup.

Figure 1 displays the technical setup for the data collection. To start and stop the recording, give instructions to the participants, and prevent technical issues, one of the experimenters attended the call with his camera and microphone turned off. In the email, participants were given the following set-up instructions: (1) to use a personal computer or laptop endowed with a camera; (2) to place the device so that the webcam was at the participant's eye level; (3) to install Zoom and create a personal account; (4) to have a plate of pasta cooked beforehand (no constraints on the sauce) and to place the plate in front of them; (5) to make sure their head was fully visible in the frame; (6) to make sure not to have windows behind; (7) to tie their hair so it would not cover their face and ears; (8) not to wear glasses, if possible; (9) to avoid headphones.

### 3.4. Questionnaires and open questions

Before the shared meal, we asked participants to complete a form collecting demographic information—age range and gender—, relationship with the co-diner—friends, very close friends, best friends—, and frequency of video-chats in their daily lives. Moreover, we asked them to respond to a questionnaire assessing their attitudes toward video-chats. To this aim, we selected and translated (see Table 2) items from the Computer Mediated Communication Questionnaire (Yen and Tu, 2008).

**Table 2:** Computer mediated communication questionnaire.

**Question**	**Please indicate the extent to which you agree or disagree with the following statement:**
*CMCQ*1	CMC allows me to perform social interactions
*CMCQ*2	CMC allows me to carry on informal conversations
*CMCQ*3	I am comfortable using CMC to communicate with a single individual or multiple people
*CMCQ*4	It is difficult to express what I want to communicate through CMC
*CMCQ*5	CMC communication becomes easier as I become more experienced in its use
*CMCQ*6	CMC allows me to build more caring social relationships with others
*CMCQ*7	CMC permits the building of trust relationships

All items were 5 point Likert-scale items ranging from strongly disagree to strongly agree.

When the shared meal was over, we asked participants to complete the final questionnaire. This step was aimed at investigating the Digital Commensality experience. To do so, participants responded to items from the Digital Commensality Questionnaire to investigate their opinion on the effects of social interaction on the eating experience, in terms of food liking, sense of belonging, feelings of loneliness, and boredom. Table 3 illustrates the Digital Commensality Questionnaire items used in this study, translated into English. As these items assess participants' opinions on Digital Commensality in general, i.e., without referring to a specific commensal experience, we added two extra items for this study (DCQ7 ad DCQ8) to gather information on participants' satisfaction with the Digital Commensality experience recorded in the videos. Thus, one can also correlate the video recordings with the corresponding levels of user (commensal) satisfaction.

**Table 3:** Digital Commensality Questionnaire items and Digital Commensality experience ratings.

**Question**	**Please indicate the extent to which you agree or disagree with statements Q1-Q6 and provide a rating to Q7-Q8:**
*DCQ*1	Eating or drinking with others online helps me feel closer to them
*DCQ*2	Eating or drinking with others online makes our meet-up more interesting
*DCQ*3	Eating or drinking with others online makes our meet-up more fun
*DCQ*4	Eating or drinking with others online helps me feel as if we were actually together
*DCQ*5	Eating or drinking with others online helps me feel less alone
*DCQ*6	Eating or drinking with someone else online makes me appreciate my food more
*DCQ*7	Overall, how would you rate the digital commensality experience?
*DCQ*8	Compared with eating in person with the same person, how would you rate your digital commensality experience?

DCQ items 1 to 6 were 5 points Likert-scale items ranging from completely disagree to completely agree. DCQ items 7 and 8 were 5 points Likert-scale items ranging from completely negative to completely positive.

To better grasp participants' opinions on the remote meals, we asked them to indicate, among a list of options, possible negative aspects of the experience. Options were the following: network problems, hearing the noise of the commensal chewing, seeing the commensal chewing, being watched while chewing, being unable to share smell or taste with the other person, communication problems (e.g., talking at the same time), personal dislike toward video-calls, and none. Lastly, participants were asked, through an open-ended question, to describe the best possible technology for digital commensality they could envision.

All the questionnaires we administered to the participants were translated into Italian and were anonymous.

### 3.5. Video recordings and facial expressions

In total, we recorded 22 participants. The total length of the recordings is 3 h and 37 min. The average duration of a single meal is 10 min and 50 s (with the shortest meal lasting 5 min and 18 s and the longest one lasting 16 min and 17 s).

**Figure 2:**
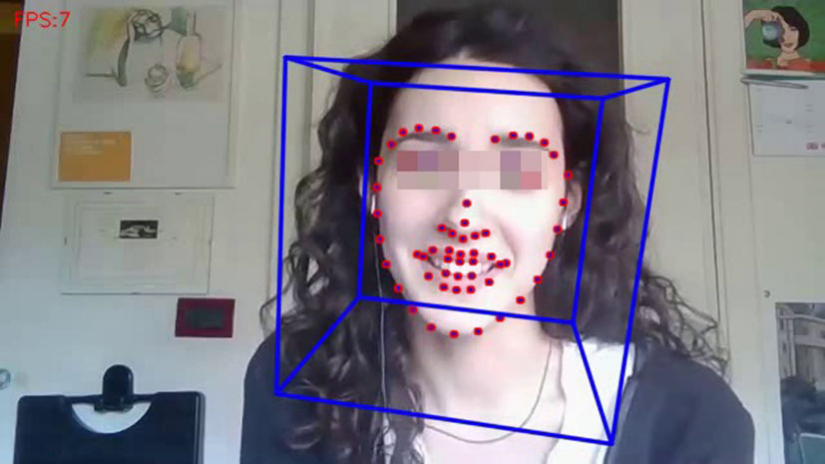
A frame of a video in the data-set, showing automated facial features extraction through OpenFace.

We automatically extracted facial features (e.g., the value of facial Action Units, Ekman and Friesen, 1978) through the state-of-the-art face tracking software OpenFace (Baltrusaitis et al., 2016), see an example frame in Figure 2. We decided to test the possibility of performing automatic extraction of facial features on our videos for two main reasons: firstly, facial features are critical in Computational Commensality, for instance, to automatically detect eating-related behaviors (chewing, chatting, food in-taking, and so on) for future implementation, e.g., in an Artificial Commensal Companion (Mancini et al., 2020); second, extracting these features on our videos helped us validate the possibility to use remotely collected videos, in our case recorded through Zoom. In our data collection, we demonstrated this approach to be viable. When we estimated this possibility by computing the number of frames dropped in the facial features extraction process, our results showed that only 4% of the frames were dropped (on average) per participant. Only for one participant, this result was worst (38% of dropped frames). By reviewing this participant's video, we noticed that the participant's webcam was slightly above his head, and he tended to tilt his head forward to eat the food (instead of raising his fork). Consequently, his face was not visible during food intake. This limitation highlights possible issues in recording commensal scenarios and provides suggestions for future recordings, e.g., being very careful of the camera setup, informing participants, and choosing food that does not require cutlery.

## 4. Analysis and results

### 4.1. Self-reported measures

We carried out all data analysis using Jasp^2^ (JASP Team, 2022). We began by assessing the internal consistency of items from the CMC and DC questionnaires through Cronbach's Alpha. Internal consistency was always high, measuring 0.78 for CMC and 0.87 for DC. As shown by Table 4, we computed the mean score and mode for each 5-points Likert scale item of the Computer-Mediated Communication questionnaire and the Digital Commensality Questionnaire.

**Table 4:** Mean score, standard deviation, and mode for each item of the Computer-Mediated Communication Questionnaire (left) and Digital Commensality Questionnaire (right).

**Question**	**Mean (s.d.)**	**Mode**	**Question**	**Mean (s.d.)**	**Mode**
CMCQ1	4.043 (0.767)	4	DCQ1	4.136 (0.990)	5
CMCQ2	3.956 (0.877)	4	DCQ2	3.727 (1.120)	4
CMCQ3	3.478 (1.122)	4	DCQ3	3.772 (1.195)	4
CMCQ4	2.695 (0.973)	3	DCQ4	3.590 (0.908)	4
CMCQ5	4.130 (0.757)	4	DCQ5	4.181 (0.852)	5
CMCQ6	3.695 (0.875)	3	DCQ6	3.227 (1.306)	4
CMCQ7	3.347 (1.112)	3	DCQ7	3.863 (0.774)	4
			DCQ8	2.636 (0.847)	3

To investigate whether other factors could explain ratings provided to the Digital Commensality Questionnaire, we explored, by measuring Spearman's statistics, the correlation between the DC items scores and, respectively, CMC items, videocalls frequency (both for work and to meet close ones) and frequency of in-person meetings with the commensal. In our analysis, none of these factors seemed to be significantly correlated (*p*>0.05) with the DC items investigating participants' opinions on their Digital Commensality experience. Table 5 illustrates the results of the correlation analysis.

**Table 5:** Spearman's correlations *r*_(20)_ with Digital Commensality items mean scores.

**Factor**	**Spearman's rho**	***p*** **-value**
CMC	0.067	0.767
frequency of videochats for work	−0.259	0.244
frequency of videochats to meet friends	−0.339	0.123
frequency of in person meetings	−0.185	0.410

**Figure 3:**
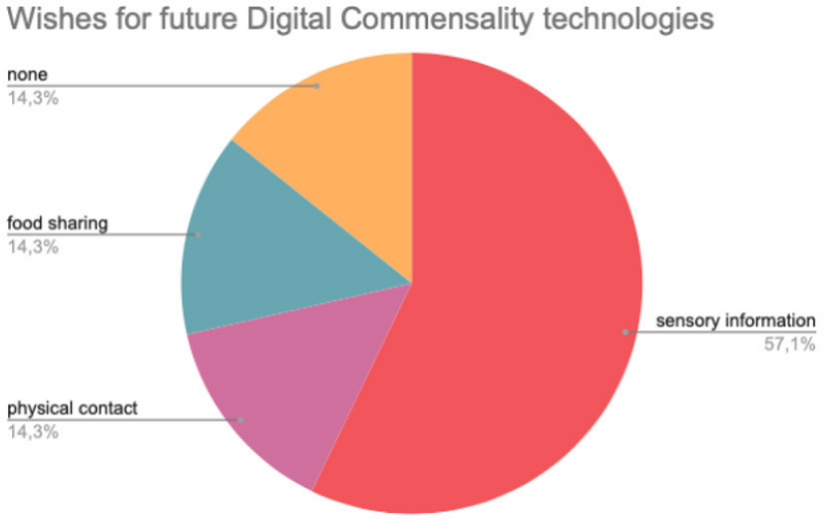
Wishes for future Digital Commensality technologies.

Moreover, we analyzed qualitative responses as they can help shed light on the negative side of the commensal experiences and can act as sources of inspiration for the design of future Digital Commensality technologies. When asked about the main downsides of their Digital Commensality experience, 37% of participants chose the impossibility of sharing food, taste, and smell; 29% reported feeling observed as they were eating, while 25% reported problems with the device or with their internet connection. We also asked participants to picture the future of Digital Commensality technologies and to indicate, if they wanted to, what such technologies should afford, independently from current technological possibilities. Since responses were diverse, as the participant could put down anything they wanted, the answers were clustered into 4 categories, as shown by Table 6. Figure 3 shows percentages for each categories.

**Table 6:** Wishes for future Digital Commensality technologies.

**Category**	**Example**
food sharing	*It would be great to taste each other's food*
physical contact	*Future technologies should remove all the barriers that remind you of being in two different places*
sensory information	*I'd love to be able to share smell and smell the other person's food*
none	The participant provided no answer

### 4.2. Action units

For each participant, we measured mean Action Unit activation intensity for each Action Unit, thus providing information on the overall facial movements observed in the videos. Tables 7–9 show the correlation between Action Units mean activation intensity and participants' ratings of the experience, as measured through items 7 and 8 from the Digital Commensality Questionnaire and through their mean. Our analysis revealed a weak, yet statistically significant, negative correlation between responses to DC item 7 and Action Unit number 4 [*r*_(20)_ = −0.384, *p* = 0.047], and a moderate negative correlation with Action Unit number 5 [*r*_(20)_ = −0.435, *p* = 0.028]. All results are illustrated in Table 7. As Table 8 shows, we also found responses to DC item 8 to be moderately correlated with Action Unit 6 [*r*_(20)_ = −0.473, *p* = 0.017] and with Action Unit 10 [*r*_(20)_ = −0.421, *p* = 0.032]. Also, scores were moderately correlated with Action Unit 4 [*r*_(20)_ = −0.593, *p* = 0.003].

**Table 7:** Spearman's correlations *r*_(20)_ with users' ratings to DC item 7.

**Action unit**	**AU1**	**AU2**	**AU4**	**AU5**	**AU6**	**AU7**	**AU9**	**AU10**	**AU12**
Spearman's rho	−0.193	−0.081	−0.384*	−0.435*	−0.251	−0.084	0.168	−0.003	−0.221
*p*-value	0.207	0.366	0.047	0.028	0.143	0.362	0.761	0.495	0.175
**Action unit**	**AU14**	**AU15**	**AU17**	**AU20**	**AU23**	**AU25**	**AU26**	**AU45**	
Spearman's rho	−0.100	−0.120	−0.218	−0.242	0.086	0.110	−0.319	−0.199	
*p*-value	0.337	0.308	0.177	0.152	0.640	0.678	0.085	0.201	

Statistical significance of the correlations is reported *via* * (*p < 0.05, **p < 0.005, ***p < 0.001).

**Table 8:** Spearman's correlations *r*_(20)_ with users' ratings to DC item 8.

**Action unit**	**AU1**	**AU2**	**AU4**	**AU5**	**AU6**	**AU7**	**AU9**	**AU10**	**AU12**
Spearman's rho	−0.080	−0.185	−0.593**	0.043	−0.473*	−0.195	0.289	−0.421*	−0.201
*p*-value	0.368	0.218	0.003	0.572	0.017	0.205	0.891	0.032	0.197
**Action unit**	**AU14**	**AU15**	**AU17**	**AU20**	**AU23**	**AU25**	**AU26**	**AU45**	
Spearman's rho	−0.337	0.012	0.124	−0.021	0.383	0.376	−0.075	−0.185	
*p*-value	0.051	0.519	0.698	0.466	0.952	0.949	0.376	0.218	

Statistical significance of the correlations is reported *via* * (*p < 0.05, **p < 0.005, ***p < 0.001).

**Table 9:** Spearman's correlations *r*_(20)_ with the mean scores of DC items 7 and 8, taken as an overall indicator of users' experience.

**Action unit**	**AU1**	**AU2**	**AU4**	**AU5**	**AU6**	**AU7**	**AU9**	**AU10**	**AU12**
Spearman's rho	−0.120	−0.123	−0.563**	−0.114	−0.424*	−0.173	0.250	−0.280	−0.193
*p*-value	0.306	0.302	0.005	0.317	0.031	0.233	0.857	0.116	0.207
**Action unit**	**AU14**	**AU15**	**AU17**	**AU20**	**AU23**	**AU25**	**AU26**	**AU45**	
Spearman's rho	−0.258	−0.019	−0.023	−0.100	0.270	0.314	−0.190	−0.166	
*p*-value	0.136	0.468	0.461	0.338	0.875	0.911	0.212	0.242	

Statistical significance of the correlations is reported *via* * (*p < 0.05, **p < 0.005, ***p < 0.001).

When we computed correlations between activation intensities of Action Units and mean scores from DC items 7 and 8 (taken as an overall indicator of users' experience), we observed a moderate negative correlation with Action Unit 4 [*r*_(20)_ = −0.563, *p* = 0.005], along with a moderate negative correlation with Action Unit 6 [*r*_(20)_ = −0.424, *p* = 0.031].

## 5. Discussion and conclusion

In this paper, we illustrated a study on Digital Commensality and a data-set of remote meals, the Digital Commensality Data-set. The data-set contains video and audio recordings of 11 pairs sharing a meal online through the Zoom videoconferencing software, automatically extracted facial features, and self-reported qualitative and qualitative measures of the commensal experience. The data-set, we believe, offers many possible ways to look at commensal behavior.

Whereas, previous work (Ceccaldi et al., 2020) had explored general opinions on Digital Commensality, this study assessed participants' attitudes and views on actual commensal experiences right after their ending. It also examined whether Digital Commensality leads to a sense of togetherness and belonging, thus positively affecting the eating experience (RQ1). Results of the Digital Commensality questionnaire seem to confirm that sharing food adds something to the social interaction and can create a sense of togetherness, even when the social interaction only occurs remotely. We found no statistically meaningful correlation when we analyzed the data to explore whether personal attitudes toward communicating through videochats and the frequency of videochats in daily lives may have contributed to the results. Overall, participants rated the Digital Commensality experience positively, although, when asked to compare it with in-person commensality (DCQ8), the most common response was neutral.

Moreover, we explored participants' opinions on the negative sides of their experience and their take on future Digital Commensality technologies (RQ2). Participants could indicate some shortcomings of currently available technologies when asked about the downsides of sharing a meal through videoconference. Mainly, they reported discomfort with being observed as they were eating and network-related problems, such as delays. Also, they lamented the impossibility of sharing food, taste, and smell. The possibility to convey sensory information was also what most participants suggested when asked to express their wishes for the future, still developing Digital Commensality technologies. They also reported wishing for future technologies to afford to share food and physical contact (for instance, as one participant suggested, through haptic illusions). As Spence and colleagues report (Spence et al., 2017), despite being associated with technical challenges, the digitization of smell has gained a lot of attention in Human-Computer Interaction research. We argue that most limitations of current Digital Commensality experiences come from the fact that the technologies that we use, for instance, to share a meal, were not explicitly designed to serve this purpose. For example, a computer camera fixed on somebody speaking might be extremely useful in a work meeting but, as our participants said, extremely annoying when that person is chewing.

In this work, we also looked at Digital Commensality through a computational approach, investigating whether we can leverage facial features to explore users' experience of commensality (RQ 3). We believe this approach comes with specific challenges: we recorded users while eating, possibly affecting the tracking of lower facial features. We only found statistically meaningful correlations with questionnaire items regarding user experience for upper-face landmarks. More specifically, Action Units corresponding to landmarks associated with the expression of negative emotions negatively correlated with positive evaluations of the experience. This result suggests the feasibility of leveraging facial expressions to investigate commensal experiences, although one should take these results with caution given the complexity of inferring emotion and affective states from facial expressions (Barrett et al., 2019; Girard et al., 2021). Nonetheless, the Digital Commensality Data-set provides data (i.e., facial landmarks) on spontaneous expressions, collected in ecological settings, as opposed to affective research, which is often based on acted facial expressions (Barrett et al., 2019).

Going back to what was on our plate, in terms of research questions, the Digital Commensality study and data collection presented here suggest the feasibility of investigating Digital Commensality through a computational approach. Although with the limitations of possible lack of experimental control and despite the technical difficulties that may arise, for instance, due to different lighting conditions, this approach has the great benefit of ensuring ecological validity, as participants can take part from their own homes. Through this approach, we built the first, to the best of our knowledge, Digital Commensality Data-set of video-recorded commensal experiences. The data collection also allowed us to gather information on the effects of (virtually) sharing a meal on social interaction, as measured by the Digital Commensality questionnaire (Ceccaldi et al., 2020), and on our participants' opinions on current and future Digital Commensality technologies. We believe our efforts can foster further studies on Digital (and real-life) commensality to shed more light on this cherished form of social interaction.

## Data availability statement

The raw data supporting the conclusions of this article will be made available by the authors, without undue reservation.

## Ethics statement

Ethical review and approval was not required for the study on human participants in accordance with the local legislation and institutional requirements. The patients/participants provided their written informed consent to participate in this study.

## Author contributions

All authors listed have made a substantial, direct, and intellectual contribution to the work and approved it for publication.

## Conflict of interest

The authors declare that the research was conducted in the absence of any commercial or financial relationships that could be construed as a potential conflict of interest.

## Publisher's note

All claims expressed in this article are solely those of the authors and do not necessarily represent those of their affiliated organizations, or those of the publisher, the editors and the reviewers. Any product that may be evaluated in this article, or claim that may be made by its manufacturer, is not guaranteed or endorsed by the publisher.

## Footnotes

1 The Digital Commensality data-set is available at the following link: https://doi.org/10.5281/zenodo.6768641.

2 The significance threshold for all the tests in this study was set at 0.05.
